# Supplementary material for: HIVprotI: an integrated web based platform for prediction and design of HIV proteins inhibitors
Source: J Cheminform. 2018 Mar 9;10:12. doi: 10.1186/s13321-018-0266-y (PMC5845081; doi:10.1186/s13321-018-0266-y)
Supplement: Supplementary file 2 — Additional file 2. Source code of HIVProtI web server. [file 13321_2018_266_MOESM2_ESM.zip › HIVprotI_Source-code/hivprotI/weka-3-6-6/doc/weka/core/tokenizers/CharacterDelimitedTokenizer.html]

CharacterDelimitedTokenizer


---


|  |  |  |
| --- | --- | --- |
| **Overview** \| **Package** \| **Class** \| **Tree** \| **Deprecated** \| **Index** \| **Help** \| **Weka's home** | |  |
| **PREV CLASS**   **NEXT CLASS** | **FRAMES**    **NO FRAMES**     **All Classes** |  |
| SUMMARY: NESTED \| FIELD \| CONSTR \| METHOD | DETAIL: FIELD \| CONSTR \| METHOD |  |


---


## weka.core.tokenizers Class CharacterDelimitedTokenizer

```
java.lang.Object
  weka.core.tokenizers.Tokenizer
      weka.core.tokenizers.CharacterDelimitedTokenizer
```

**All Implemented Interfaces:**: java.io.Serializable, java.util.Enumeration, OptionHandler, RevisionHandler

**Direct Known Subclasses:**: NGramTokenizer, WordTokenizer

---

``` public abstract class CharacterDelimitedTokenizer extends Tokenizer ```

Abstract superclass for tokenizers that take characters as delimiters.

**Version:**
:   $Revision: 1.3 $

**Author:**
:   fracpete (fracpete at waikato dot ac dot nz)

**See Also:**: Serialized Form

---

**Constructor Summary**

`CharacterDelimitedTokenizer()`


| **Method Summary** | |
| --- | --- |
| `java.lang.String` | `delimitersTipText()`             Returns the tip text for this property |
| `java.lang.String` | `getDelimiters()`             Get the value of delimiters (not backquoted). |
| `java.lang.String[]` | `getOptions()`             Gets the current option settings for the OptionHandler. |
| `java.util.Enumeration` | `listOptions()`             Returns an enumeration of all the available options.. |
| `void` | `setDelimiters(java.lang.String value)`             Set the value of delimiters. |
| `void` | `setOptions(java.lang.String[] options)`             Sets the OptionHandler's options using the given list. |

**Methods inherited from class weka.core.tokenizers.Tokenizer**

`globalInfo, hasMoreElements, nextElement, runTokenizer, tokenize, tokenize`

**Methods inherited from class java.lang.Object**

`equals, getClass, hashCode, notify, notifyAll, toString, wait, wait, wait`

**Methods inherited from interface weka.core.RevisionHandler**

`getRevision`

**Constructor Detail**

### CharacterDelimitedTokenizer

```
public CharacterDelimitedTokenizer()
```


**Method Detail**

### listOptions

```
public java.util.Enumeration listOptions()
```

:   Returns an enumeration of all the available options..

    :   **Specified by:**: `listOptions` in interface `OptionHandler` **Overrides:**: `listOptions` in class `Tokenizer`
    :   **Returns:**: an enumeration of all available options.

---


### getOptions

```
public java.lang.String[] getOptions()
```

:   Gets the current option settings for the OptionHandler.

    :   **Specified by:**: `getOptions` in interface `OptionHandler` **Overrides:**: `getOptions` in class `Tokenizer`
    :   **Returns:**: the list of current option settings as an array of strings

---


### setOptions

```
public void setOptions(java.lang.String[] options)
                throws java.lang.Exception
```

:   Sets the OptionHandler's options using the given list. All options
    will be set (or reset) during this call (i.e. incremental setting
    of options is not possible).

    :   **Specified by:**: `setOptions` in interface `OptionHandler` **Overrides:**: `setOptions` in class `Tokenizer`
    :   **Parameters:**: `options` - the list of options as an array of strings **Throws:**: `java.lang.Exception` - if an option is not supported

---


### getDelimiters

```
public java.lang.String getDelimiters()
```

:   Get the value of delimiters (not backquoted).

    :   **Returns:**: Value of delimiters.

---


### setDelimiters

```
public void setDelimiters(java.lang.String value)
```

:   Set the value of delimiters. For convenienve, the strings
    "\r", "\n", "\t", "\'", "\\" get automatically translated into their
    character representations '\r', '\n', '\t', '\'', '\\'. This means, one
    can either use `setDelimiters("\r\n\t\\");` or
    `setDelimiters("\\r\\n\\t\\\\");`.

    :   **Parameters:**: `value` - Value to assign to delimiters. **See Also:**: `Utils.unbackQuoteChars(String)`

---


### delimitersTipText

```
public java.lang.String delimitersTipText()
```

:   Returns the tip text for this property

    :   **Returns:**: tip text for this property suitable for displaying in the explorer/experimenter gui


---


|  |  |  |
| --- | --- | --- |
| **Overview** \| **Package** \| **Class** \| **Tree** \| **Deprecated** \| **Index** \| **Help** \| **Weka's home** | |  |
| **PREV CLASS**   **NEXT CLASS** | **FRAMES**    **NO FRAMES**     **All Classes** |  |
| SUMMARY: NESTED \| FIELD \| CONSTR \| METHOD | DETAIL: FIELD \| CONSTR \| METHOD |  |


---
